# Stereotactic Ablative Radiotherapy for Delayed Retrobulbar Metastasis of Renal Cell Carcinoma: Therapeutic Outcomes and Practical Insights

**DOI:** 10.3390/life15081176

**Published:** 2025-07-24

**Authors:** Sang Jun Byun, Byung Hoon Kim, Seung Gyu Park, Euncheol Choi

**Affiliations:** 1 Department of Radiation Oncology, Keimyung University School of Medicine, Daegu 42601, Republic of Korea; psk818@dsmc.or.kr (S.G.P.); cec0510@dsmc.or.kr (E.C.); 2 Department of Urology, Keimyung University School of Medicine, Daegu 42601, Republic of Korea; blackporori@dsmc.or.kr

**Keywords:** renal cell carcinoma, retrobulbar metastasis, stereotactic ablative radiotherapy, oligometastatic disease, immune checkpoint inhibitor

## Abstract

We present a rare case of delayed retrobulbar and adrenal metastases from renal cell carcinoma (RCC), diagnosed 5.5 years after radical nephrectomy. The patient exhibited symptomatic orbital involvement, with imaging revealing a hypervascular retrobulbar mass and an incidental right adrenal lesion, indicative of an oligometastatic state. Owing to the patient’s refusal of surgical resection, stereotactic ablative radiotherapy (SABR) was delivered to the retrobulbar lesion at a total dose of 40 Gy in five fractions, concurrently with immune checkpoint inhibitor therapy. Treatment planning prioritized sparing adjacent critical structures, including the optic chiasm and brainstem. Follow-up over 4 years demonstrated sustained radiologic stability and volume reduction in both metastatic lesions without evidence of progression. This case underscores the potential efficacy of SABR in achieving durable local control of RCC metastases, particularly in anatomically constrained regions where surgery is unfeasible. Moreover, it highlights the value of a multidisciplinary, multimodal treatment approach incorporating advanced radiotherapy techniques and systemic immunotherapy. Lastly, it reinforces the importance of prolonged surveillance in RCC survivors due to the potential for late metastatic recurrence at uncommon sites.

## 1. Introduction

Renal cell carcinoma (RCC) is the most common primary malignancy of the kidney in adults, accounting for approximately 90% of all renal cancers [1]. It is characterized by unpredictable biological behavior, including resistance to conventional therapies and a high propensity for hematogenous dissemination. Although the lungs, bones, liver, and brain are the most frequent metastatic sites, RCC is also known to spread to atypical locations, sometimes many years after initial curative surgery [2]. Among these, the orbit is considered one of the unusual sites for RCC metastasis. 

One published study indicated that the incidence of orbital metastasis from RCC is exceedingly low, with an incidence of around 3% [3]. Orbital metastases from systemic malignancies are rare, comprising only 2–3% of all orbital tumors [4]. Among these, breast, lung, and prostate cancers are the most common primaries, while RCC contributes only a small fraction. Within the orbit, retrobulbar space involvement without adjacent bony erosion is particularly uncommon. Among 71 reported cases of ocular and periocular metastasis from RCC, extraocular soft tissue metastasis was the most common (46.5%), followed by intraocular involvement (45.1%). In contrast, orbital (retrobulbar) metastasis was observed in only 27 cases (38.0%), highlighting its relatively uncommon occurrence compared with other sites of ocular involvement [3]. The orbit’s compact anatomy, dense vascularity, and proximity to critical visual structures make retrobulbar lesions both clinically significant and therapeutically challenging. Presenting symptoms typically include proptosis, diplopia, and visual disturbance; however, due to their rarity, such metastases often lead to diagnostic delays [5]. Treatment is complex and requires individualized approaches that balance local control with functional preservation.

Another unique feature of RCC is its potential for delayed metastasis, which can occur more than a decade after nephrectomy [6]. Although rare, late recurrences have been documented in both common and unusual sites, such as the brain, gastrointestinal tract, and soft tissues. These occurrences are thought to reflect mechanisms like tumor dormancy and immune evasion, emphasizing the need for long-term surveillance even in patients previously considered cured. 

Although RCC has long been regarded as a radioresistant malignancy due to historically poor outcomes with conventional low-dose, fractionated radiotherapy, this paradigm has shifted with the advent of advanced radiotherapy techniques. In particular, stereotactic body radiotherapy (SBRT), also known as stereotactic ablative radiotherapy (SABR), facilitates the delivery of highly conformal, ablative doses in a limited number of fractions, thereby overcoming some of the inherent resistance associated with traditional radiation schedules [7]. Emerging clinical evidence has demonstrated that SABR can provide durable, local control and symptom relief in both primary and metastatic RCC, especially in oligometastatic settings or anatomically constrained sites where surgery is not feasible or poses significant risk [8]. Furthermore, radiobiological models suggest that RCC, despite its low α/β ratio, may respond favorably to high-dose-per-fraction regimens that induce endothelial damage and promote immune modulation. As such, radiotherapy is increasingly being incorporated into multidisciplinary RCC management strategies, not only for palliation but also as a potentially curative modality in select patients [9].

We present a rare case of RCC with retrobulbar and adrenal metastases diagnosed 5.5 years after radical nephrectomy. The retrobulbar lesion was symptomatic, whereas the adrenal mass was discovered incidentally. The patient was treated with a combination of SABR to the orbit and systemic immune checkpoint inhibitor therapy. This case illustrates the role of radiotherapy as part of a multimodal approach to achieve local control in the era of immunotherapy.

## 2. Detailed Case Description

**Figure 1:**
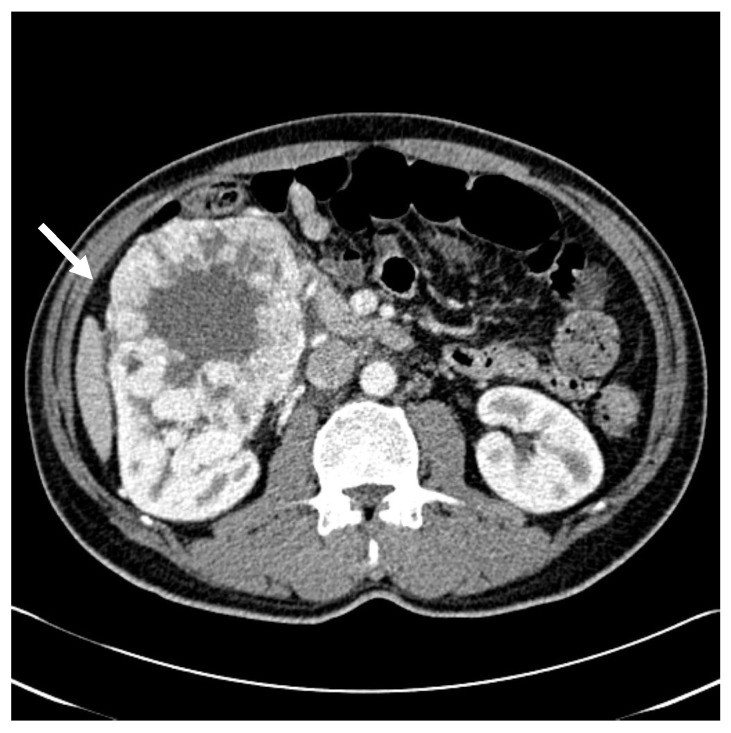
Initial abdominal CT showed a lobulated, heterogeneously and avidly enhancing mass measuring up to 14.3 cm in maximal diameter, located in the anterior portion of the right kidney’s lower pole (white arrow). The lesion exhibited internal necrosis and contained multiple calcific foci.

A 51-year-old man presented to the nephrology department of our hospital with painless gross hematuria. He was undergoing treatment for schizophrenia at a local psychiatric clinic. His medical history included hypertension, although he was not taking antihypertensive medication regularly. He reported social alcohol use and had a 10-pack-per-year smoking history. Hematological tests at the nephrology outpatient clinic were unremarkable except for abnormal findings on urinalysis. Abdominal computed tomography (CT) revealed a 10.4 × 14.3 × 10.0 cm^3^ mass in the right kidney (Figure 1), while chest CT showed no significant findings. The patient subsequently underwent radical nephrectomy of the right kidney, and histopathology confirmed clear cell carcinoma with nuclear grade III/IV. The tumor was confined to the kidney and measured 13.0 × 10.5 × 10.0 cm^3^ (pT2b). There was no evidence of renal vein invasion, and surgical margins were negative in all directions. Follow-up CT scans of the brain, chest, and abdomen at 1 and 6 months postoperatively showed no recurrence. Axitinib was initiated 6 months post-surgery but was discontinued 3 months later at the patient’s request. The patient was then lost to follow-up.

**Figure 2:**
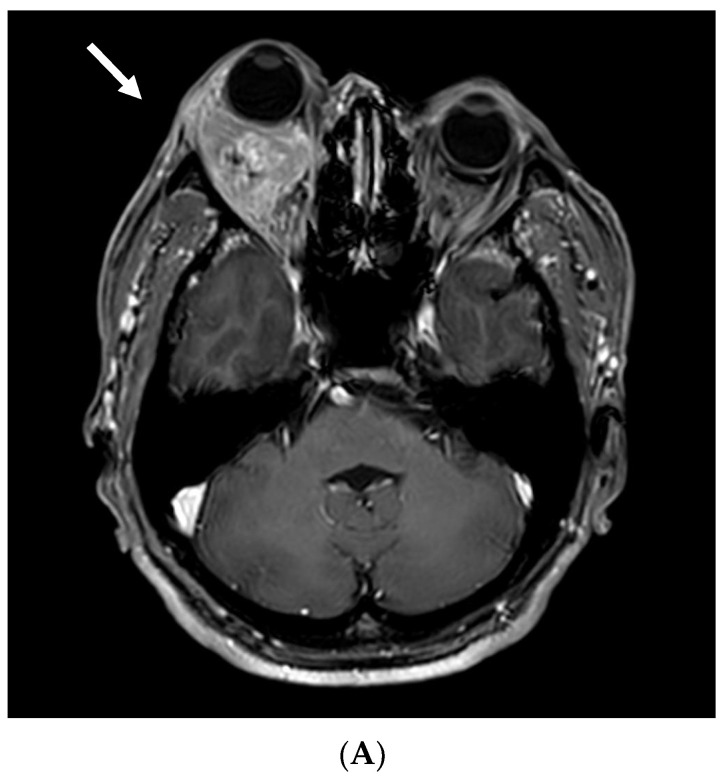

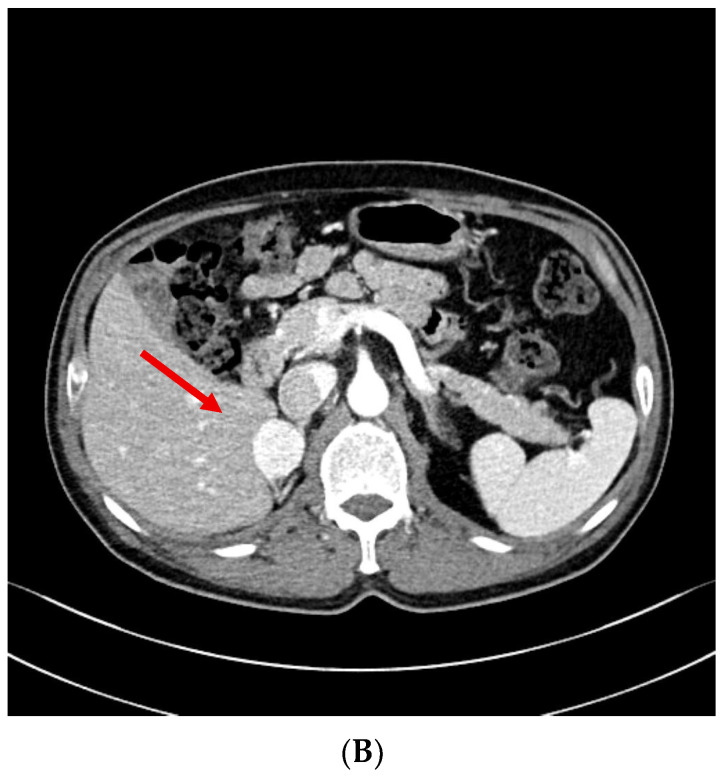
(**A**) Orbital MRI demonstrated a hypervascular retrobulbar mass in the right orbit measuring approximately 3.0 × 3.7 × 2.8 cm^3^, with multiple signal void vessels (white arrow). (**B**) Abdominal CT revealed a 2.9 × 3.0 × 2.6 cm^3^ enhancing mass in the right nephrectomy bed, suggestive of local recurrence (red arrow).

Five and a half years later, the patient was referred from a local ophthalmology clinic due to exophthalmos. Abdominal CT and orbital magnetic resonance imaging (MRI) revealed a 2.9 × 3 × 2.6 cm^3^ adrenal mass and a 3.0 × 3.7 × 2.8 cm^3^ irregularly shaped retrobulbar mass behind the right globe, respectively (Figure 2). A urologic oncologist initiated sunitinib therapy and recommended surgical resection of the orbital lesion. However, the patient declined surgery, including right eye enucleation, and was referred to a radiation oncologist for evaluation and management of the orbital mass.

**Figure 3:**
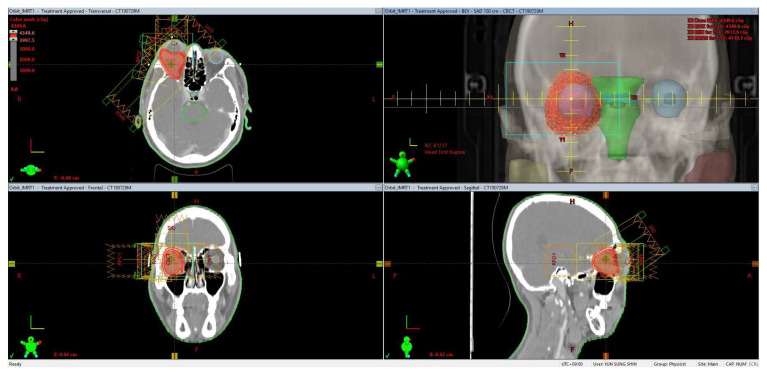
Stereotactic ablative radiotherapy dose distribution for the retrobulbar metastasis. Red areas indicate the planned prescription dose of 40 Gy delivered in five fractions.

For simulation CT, the patient was positioned supine, and the head and neck were immobilized using an Aquaplast mask. CT simulation was performed with a slice thickness of 3 mm. For treatment planning, the clinical target volume (CTV) was delineated based on MRI fusion. The planning target volume (PTV) was generated by expanding the CTV by 2–3 mm, taking into account the proximity of surrounding organs at risk (OARs). Following the organs-at-risk dose constraints outlined in the AAPM TG-101 report for 5-fraction SBRT, the treatment plan was designed to restrict the maximum dose to the optic chiasm to ≤30 Gy and to the brainstem to ≤31 Gy, thereby minimizing the potential for radiation-induced injury to these critical structures [10]. Radiation was delivered to the right retrobulbar area using the SABR technique, with a total dose of 40 Gy in five fractions, administered on alternate days (Figure 3).

**Figure 4:**
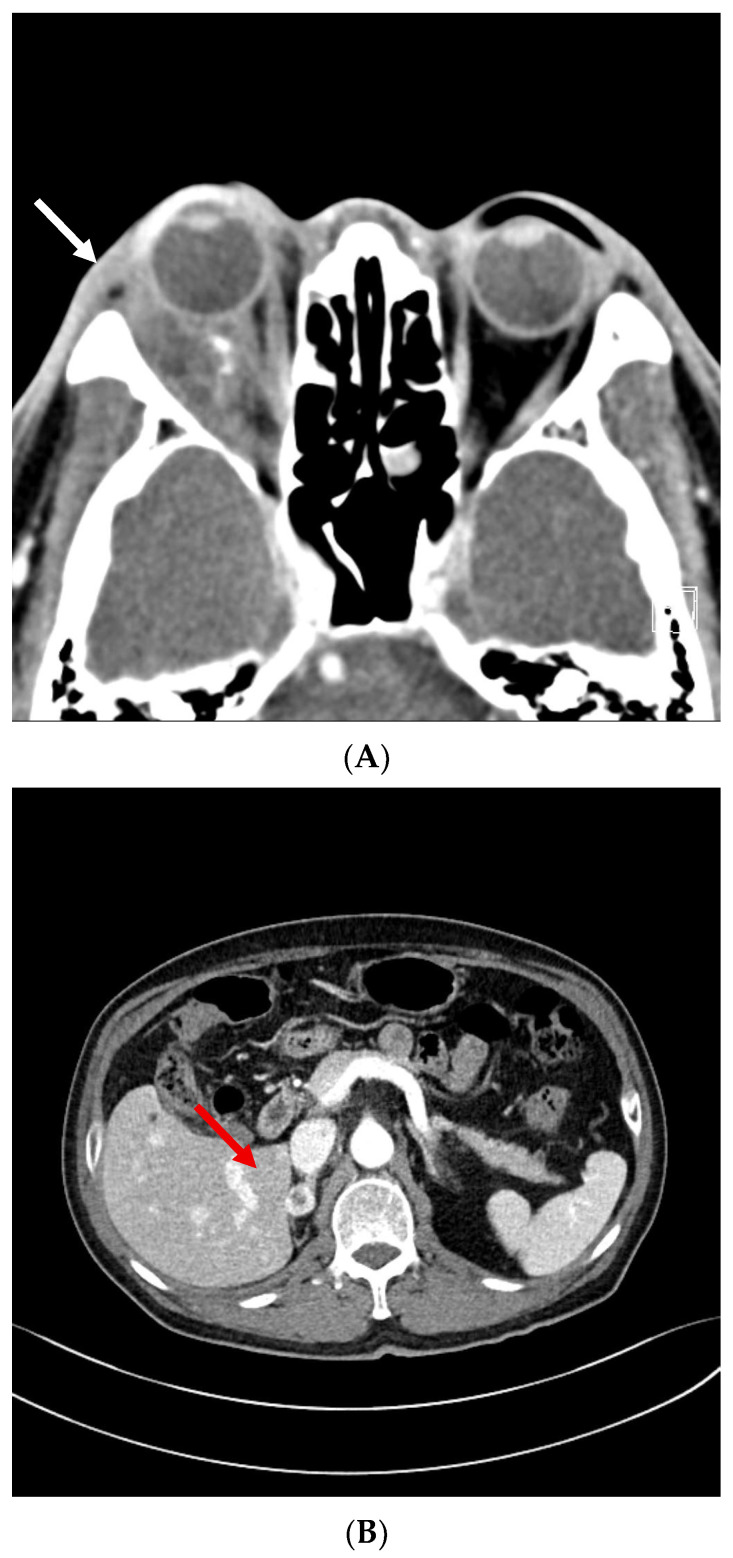
(**A**) Follow-up imaging demonstrated radiologic stability of the right retrobulbar mass, which had previously decreased in size on orbital CT (white arrow). (**B**) The right adrenal lesion also remained stable, with no progression after decreasing from 3.0 cm to 1.6 cm in maximal diameter on abdominal CT (red arrow).

**Figure 5:**
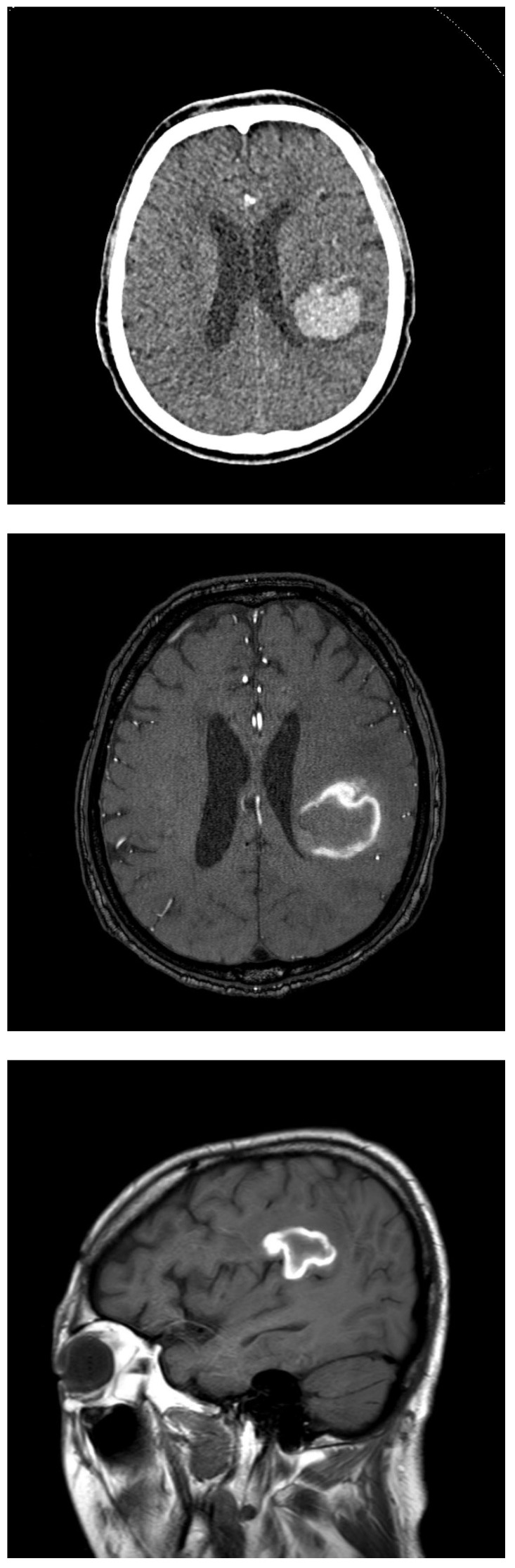
Brain CT and MRI showed findings consistent with acute infarction involving the left frontal lobe and left basal ganglia.

A 2-month follow-up MRI demonstrated a reduction in the size of the orbital mass, and a 3-month follow-up abdominal CT also revealed shrinkage of the right adrenal metastasis, which had not been treated with radiotherapy. At 7 months post-radiotherapy, an orbital MRI continued to show lesion regression. Serial imaging was conducted approximately every 6 months, and the treated lesions remained stable in size for up to 4 years following SABR (Figure 4). At 4 years and 2 months post-radiotherapy, the patient presented to the emergency department with altered mental status. Brain MRI revealed a hemorrhagic lesion in the left frontoparietal lobe, anatomically distinct from the prior radiotherapy site (Figure 5). The patient received acute-phase management and was subsequently transferred to a rehabilitation facility. A timeline table summarizing the patient’s disease course, including diagnosis, treatment interventions, and clinical follow-up, is presented in Table 1.

## 3. Discussion

We described a case of RCC that exhibited delayed metastases following radical nephrectomy, subsequently managed with SABR and immunotherapy. The patient presented with symptomatic retrobulbar metastasis 5.5 years after surgery for RCC. Concurrently, an asymptomatic adrenal metastasis was detected, representing an oligometastatic disease state. Given the orbit’s anatomical complexity and the importance of preserving visual function, surgical resection was not pursued. Instead, SABR was employed to achieve local control of the orbital lesion.

RCC is well known for its unpredictable metastatic behavior and potential for late recurrence. Hematogenous dissemination, typically via the renal vein and inferior vena cava, allows for extensive systemic spread. Although the lungs, bones, liver, and brain are common metastatic sites, RCC can also involve rare locations such as the orbit [1,2]. Orbital metastasis is an unusual manifestation of RCC and may occur either before or after the primary diagnosis. Within the orbit, the retrobulbar compartment is particularly vulnerable due to its confined anatomy and proximity to critical structures, including the optic nerve and extraocular muscles. Lesions in this space frequently cause proptosis, diplopia, pain, or visual deficits, necessitating prompt evaluation. Imaging is key for lesion localization, while histopathologic confirmation remains essential for definitive diagnosis [4,5].

Delayed metastasis is a recognized but uncommon characteristic of RCC. Numerous case reports have documented recurrence more than 10 or even 20 years post-nephrectomy, involving sites such as the brain, gastrointestinal tract, soft tissue, and breast [6,11,12,13]. The underlying biology remains poorly defined but is believed to involve mechanisms such as tumor dormancy, immune escape, and neovascularization. Dormant tumor cells may remain inactive for years and become reactivated by changes in the microenvironment or host immune modulation. This case reinforces RCC’s capacity for late relapse and highlights the need for prolonged clinical surveillance even after presumed curative treatment. This underscores the importance of long-term clinical vigilance, even in patients who remain free of recurrence for many years. Clinicians should maintain a high index of suspicion for new or atypical symptoms in RCC survivors, as these may indicate delayed metastatic disease. Several reports have emphasized the clinical relevance of such late metastases. One case described a solitary cerebral metastasis that emerged 15 years after curative nephrectomy for stage T1N0M0 RCC, illustrating that even low-stage disease can recur after prolonged latency [6]. In addition, a review of atypical late recurrences identified multiple cases involving rare sites such as the breast and thyroid, further supporting the notion that RCC can remain dormant and later reemerge in unexpected locations [14]. Collectively, these findings reinforce the need for extended follow-up and sustained clinical awareness, even long after initial treatment. In support of our case findings, a brief literature review of similar reports describing delayed metastatic presentations of RCC after nephrectomy is presented in Table 2. This summary highlights the variability in metastatic sites, latency periods, and treatment outcomes [12,14,15,16].

Importantly, the patient received SABR in combination with systemic immune checkpoint inhibitor therapy, exemplifying a multimodal approach. Radiotherapy was delivered using a hypofractionated regimen of 8 Gy per fraction, in contrast to the conventional 1.8–2 Gy per fraction schedule. This decision was based on growing evidence that RCC exhibits relative radioresistance when treated with standard fractionation [17].

Deschavanne et al. identified RCC as the most radioresistant tumor among 76 human tumor and normal cell lines studied [18]. RCC cells demonstrated the highest survival fraction after ionizing radiation exposure, reflecting their intrinsic resistance in vitro [18]. A central molecular mechanism contributing to this resistance is the abnormal stabilization and overexpression of hypoxia-inducible factor 1-alpha (HIF-1α), a transcription factor critical to cellular adaptation under hypoxia [19]. In clear cell RCC, inactivation of the von Hippel–Lindau (VHL) tumor suppressor gene leads to constitutive HIF-1α activation even in normoxia [20]. This, in turn, promotes transcription of genes involved in glycolysis, angiogenesis, cell survival, and resistance to oxidative stress, creating a tumor microenvironment highly resilient to radiation-induced cytotoxicity [21]. Building upon prior findings, a study explored the role of HIF-1α in radiation-induced bystander effects within RCC. Under hypoxic conditions during irradiation, RCC cells exhibited markedly increased expression of HIF-1α, which correlated with elevated secretion of cytokines such as TGF-β and IL-6. These factors acted on adjacent non-irradiated cells, enhancing their survival and resistance to injury—hallmarks of the bystander effect. The study suggested that HIF-1α mediates both direct responses in irradiated tumor cells and paracrine effects within the microenvironment, collectively fostering a more radioresistant tumor phenotype. These findings support the rationale for combining HIF-1α inhibitors with radiotherapy to improve local control [22]. A recent translational review explored the role of HIF-1α in mediating radioresistance and its therapeutic implications in clinical oncology. Synthesizing data from preclinical models and early-phase clinical trials, the review showed that tumors with elevated HIF-1α expression often exhibit poor radiologic response and early recurrence following radiotherapy. Mechanistically, HIF-1α was shown to promote angiogenesis via VEGF, facilitate DNA repair, and enhance epithelial–mesenchymal transition (EMT)—pathways that contribute to radiation evasion and increased metastatic potential. The review also highlighted that inhibiting HIF-1α or its downstream effectors, such as VEGF signaling or the PI3K/AKT/mTOR axis, may sensitize tumors to radiation. Early-phase trials evaluating HIF-1α inhibitors and anti-angiogenic agents in combination with radiotherapy or systemic immunotherapy were cited as promising avenues to improve treatment outcomes in RCC [23].

Advances in radiotherapy technology have facilitated a paradigm shift, allowing precise tumor targeting and delivery of higher biologically effective doses while sparing adjacent normal tissues. Ning et al. studied two human RCC cell lines, Caki-1 and A498, and found that their α/β ratios ranged from 2.6 to 6.9 Gy, substantially lower than the ~10 Gy typical of radiosensitive tumors. This radiobiologic profile suggests that RCC may respond more favorably to hypofractionated radiotherapy with higher doses per fraction [24]. Given this rationale, a dose of 8 Gy per fraction was selected for the present case, with careful consideration of adjacent organs at risk, including the optic apparatus and periorbital structures. Although the tumor was located in close proximity to critical visual structures, meticulous planning allowed for adequate target coverage while respecting dose constraints. Continued surveillance remains essential to assess potential late effects.

The use of SABR in metastatic RCC is gaining increasing recognition, as emerging clinical data continue to challenge the longstanding characterization of RCC as a radioresistant malignancy. While RCC has traditionally shown limited response to conventional fractionated radiotherapy, recent advances in high-precision techniques such as SABR have enabled new therapeutic applications, particularly in the context of oligometastatic and oligoprogressive disease. In a prospective observational cohort, SABR demonstrated strong clinical efficacy and safety in patients with limited metastatic burden. Over 90% of treated lesions remained progression-free at 1 year, and treatment was well tolerated, with minimal acute or late toxicity. Notably, lesions smaller than 14 mm in maximum diameter exhibited significantly better progression-free survival than larger lesions. These findings suggest that early detection and localized intervention using SABR may not only provide effective tumor control but also delay the need for systemic therapy, thereby preserving quality of life and minimizing treatment-related morbidity [25]. Complementary data from multi-institutional studies support these outcomes. A comprehensive review of SABR in metastatic RCC reported consistent local control rates of ≥85% across diverse metastatic sites, including lung, bone, and liver. Toxicities were predominantly low-grade and infrequent, reinforcing the view that SABR offers a favorable therapeutic ratio. These results support the role of SABR not merely as a palliative option, but as a potentially definitive treatment in selected patients with limited disease burden. Its non-invasive nature and outpatient delivery make SABR particularly appealing for elderly or medically inoperable patients who are not candidates for surgery or intensive systemic regimens [26]. The durability of response to SABR is further supported by long-term data. A multicenter study comparing single-fraction versus multi-fraction SABR found that single high-dose treatment was associated with superior local control and reduced retreatment rates. This benefit may reflect the ablative potential of high-dose radiation to overcome RCC’s intrinsic radioresistance. The study also underscored the importance of careful patient selection and personalized treatment planning, highlighting that dosimetric parameters and lesion-specific factors must be integrated into clinical decision-making to optimize therapeutic outcomes [27]. Expanding the therapeutic scope of SABR, a recent literature review outlined its growing role within the evolving RCC treatment paradigm. The review emphasized that SBRT is now routinely used in cases of oligoprogressive disease to ablate resistant clones while allowing systemic therapy to continue controlling other metastatic sites. Additionally, SABR is under active investigation as a cytoreductive modality, either prior to or alongside systemic immunotherapy. Early clinical data suggest that radiation-induced tumor antigen release and modulation of the tumor microenvironment may enhance immune responsiveness, providing a biological rationale for combining SABR with immunotherapy. This emerging synergy positions SBRT as a central component of future RCC treatment strategies [28]. Notably, during follow-up imaging, shrinkage of the right adrenal metastasis—which had not been targeted by radiotherapy—was observed after SABR to the intraorbital lesion and concurrent administration of immune checkpoint inhibitors. This raises the possibility of an abscopal effect, whereby localized radiation may have triggered a systemic antitumor immune response. Although we cannot definitively attribute the regression to this mechanism without immunologic correlates, the temporal association supports a potential synergistic interaction between SABR and immunotherapy. Further support for combination approaches comes from studies evaluating SABR with tyrosine kinase inhibitors (TKIs), a mainstay of systemic therapy in advanced RCC. In one such study, patients who received SABR in addition to TKI therapy demonstrated significantly improved clinical outcomes compared to those treated with TKIs alone. Specifically, the addition of SABR resulted in prolonged overall survival and higher complete response rates, especially in patients treated with local therapy prior to systemic progression. These findings suggest that early integration of SABR may delay TKI resistance, augment systemic control by eliminating residual tumor burden, and potentially allow for treatment holidays. This evidence advocates for a proactive, rather than reactive, use of local therapy, positioning SABR as a tool for consolidating systemic responses and extending durable disease control [29].

Collectively, this expanding body of literature underscores the growing utility of SABR in the management of metastatic RCC. From achieving durable local control with minimal toxicity to synergizing with systemic agents, SABR represents a versatile tool within the multidisciplinary treatment armamentarium. Continued prospective investigation is warranted to refine optimal timing, patient selection, and integration strategies within evolving RCC treatment algorithms. Both preclinical and clinical studies have demonstrated that higher fractional doses may be more effective in overcoming the intrinsic radioresistance of RCC cells [30]. Hypofractionated radiotherapy, including SABR, has been associated with favorable local control in RCC metastases, particularly at osseous and pulmonary sites [31]. In this context, our use of 8 Gy per fraction was intended to enhance tumoricidal efficacy while minimizing toxicity to adjacent orbital structures. Additionally, radiotherapy has been shown to exert immunomodulatory effects, such as enhanced antigen presentation and dendritic cell activation, which may synergize with immune checkpoint inhibitors to generate both local and systemic responses, a phenomenon termed the abscopal effect [32]. Although rare, the abscopal effect has been observed in RCC and other solid tumors treated with radiotherapy and immunotherapy.

SABR has emerged as a promising local treatment modality for metastatic lesions located in anatomically challenging or surgically inaccessible sites, offering high precision and a short overall treatment duration. However, its implementation may be limited by the high cost of equipment, the need for specialized personnel, and restricted availability in certain healthcare settings. These barriers can lead to disparities in access, particularly in resource-constrained institutions. Future clinical research and health policy efforts should focus on evaluating the cost-effectiveness of SABR and developing strategies to improve its accessibility across diverse clinical environments.

This case contributes to the limited but growing literature supporting the role of SABR in managing orbital RCC metastases and underscores the clinical relevance of hypofractionation in radioresistant tumors. It also illustrates the practical integration of local and systemic therapies in the immuno-oncology era. Finally, it reinforces the need for prolonged surveillance in RCC survivors and a broad differential diagnosis in patients presenting with atypical ophthalmologic symptoms. Nevertheless, several limitations are inherent to this case report. Most notably, the concurrent administration of immune checkpoint inhibitor therapy and SABR precludes attribution of the observed clinical response to either modality alone. While the tumor regression and symptom relief may suggest synergistic interaction, possibly mediated by an abscopal mechanism, this cannot be confirmed without controlled studies or immunologic correlative analyses. Moreover, the relatively short follow-up period limits assessment of long-term local control and systemic progression. As with all single-case reports, these findings should be interpreted with caution, and further prospective studies are needed to validate the efficacy of combined modality treatment in similar clinical scenarios. 

## 4. Conclusions

This case highlights the rare presentation of retrobulbar metastasis from RCC, diagnosed 5.5 years after initial nephrectomy and concurrently with an asymptomatic adrenal lesion. It underscores the importance of including RCC in the differential diagnosis of orbital masses, even in patients with a remote oncologic history. The favorable clinical response observed following hypofractionated EBRT in combination with systemic immune checkpoint inhibition suggests that a multimodal approach may be effective for achieving local control in anatomically constrained sites such as the orbit. A multidisciplinary approach—encompassing urology, medical oncology, and radiation oncology—is essential to optimize both diagnostic accuracy and therapeutic outcomes in such complex cases. Further investigation is warranted to validate the efficacy, durability, and safety of such combined treatment strategies in patients with metastatic RCC involving uncommon sites. Prospective clinical studies, comprehensive registry data, or investigations incorporating immunological biomarkers are needed to better define optimal multimodal management strategies for metastatic RCC to rare anatomical locations.

## Figures and Tables

**Table 1:** Timeline of treatments and the clinical course.

Time Point	Treatment	Key Clinical Events
Initial diagnosis		Initial presentation with gross hematuria Abdominal CT: 10.4 × 14.3 × 10.0 cm^3^ right renal mass
	Surgery	Radical nephrectomy performed Pathology: clear cell RCC, Grade III/IV, pT2b
1 and 6 months after surgery		Follow-up CT of brain, chest, and abdomen: No recurrence
6 months after surgery	axitinib initiated	
9 months after surgery	axitinib discontinued at patient’s request → Lost to follow-up	
5.5 years after surgery		Referred due to exophthalmos → CT/MRI: Right adrenal (2.9 × 3.0 × 2.6 cm^3^) and right retrobulbar (3.0 × 3.7 × 2.8 cm^3^) metastases
	sunitinib initiated → Surgical resection recommended but declined → SABR (40 Gy in 5 fractions)	
2 months after SABR	Continued sunitinib	Decrease in orbital lesion size (2.1 × 3.0 × 2.2 cm^3^) on MRI
3 months after SABR	Continued sunitinib	Decrease in adrenal metastasis size (1.6 × 1.4 × 1.5 cm^3^) on CT
7 months after SABR	Continued sunitinib	Continued regression of orbital lesion (1.9 × 1.8 × 1.7 cm^3^)
Up to 4 years after SABR	Continued sunitinib	Stable disease confirmed on serial imaging every 6 months
4 years 2 months after SABR		Acute mental status change → Brain MRI: Hemorrhagic lesion in left frontoparietal lobe (anatomically distinct from the prior SABR)
After acute care		Transferred to rehabilitation facility

**Table 2:** Representative case reports describing delayed metastasis of renal cell carcinoma following nephrectomy.

Author (Year)	Site of Metastasis	Interval After Nephrectomy	Treatment	Outcome and Significance
Marra et al. (2023) [12]	Scalp soft tissue	27 years	Surgical resection + histopathologic confirmation	Demonstrates the potential for extremely delayed RCC metastasis to soft tissue.
Lou et al. (2023) [15]	Pancreas	16 years	Pancreaticoduodenectomy (Whipple)	Isolated pancreatic metastasis from RCC can occur after long latency; surgery is effective.
Khalafi-Nezhad et al. (2024) [14]	Thyroid gland	13 years	Thyroidectomy + immunohistochemistry	Late-onset thyroid nodules can be first indicator of RCC metastasis.
Magara et al. (2024) [16]	Stomach (submucosal)	12 years	Full-thickness gastric resection	Rare gastric metastasis; surgical management led to favorable prognosis.

## Data Availability

The raw data that support the findings of this study are available from the corresponding author upon reasonable request. However, the data are not publicly available due to privacy considerations.
